# Identifying Redox-Sensitive Cysteine Residues in Mitochondria

**DOI:** 10.3390/antiox12050992

**Published:** 2023-04-25

**Authors:** Eleni A. Kisty, Emma C. Saart, Eranthie Weerapana

**Affiliations:** Department of Chemistry, Boston College, Chestnut Hill, MA 02467, USA

**Keywords:** mitochondria, cysteine, ROS, oxidation, mass spectrometry, isoTOP-ABPP, OxICAT

## Abstract

The mitochondrion is the primary energy generator of a cell and is a central player in cellular redox regulation. Mitochondrial reactive oxygen species (mtROS) are the natural byproducts of cellular respiration that are critical for the redox signaling events that regulate a cell’s metabolism. These redox signaling pathways primarily rely on the reversible oxidation of the cysteine residues on mitochondrial proteins. Several key sites of this cysteine oxidation on mitochondrial proteins have been identified and shown to modulate downstream signaling pathways. To further our understanding of mitochondrial cysteine oxidation and to identify uncharacterized redox-sensitive cysteines, we coupled mitochondrial enrichment with redox proteomics. Briefly, differential centrifugation methods were used to enrich for mitochondria. These purified mitochondria were subjected to both exogenous and endogenous ROS treatments and analyzed by two redox proteomics methods. A competitive cysteine-reactive profiling strategy, termed isoTOP-ABPP, enabled the ranking of the cysteines by their redox sensitivity, due to a loss of reactivity induced by cysteine oxidation. A modified OxICAT method enabled a quantification of the percentage of reversible cysteine oxidation. Initially, we assessed the cysteine oxidation upon treatment with a range of exogenous hydrogen peroxide concentrations, which allowed us to differentiate the mitochondrial cysteines by their susceptibility to oxidation. We then analyzed the cysteine oxidation upon inducing reactive oxygen species generation via the inhibition of the electron transport chain. Together, these methods identified the mitochondrial cysteines that were sensitive to endogenous and exogenous ROS, including several previously known redox-regulated cysteines and uncharacterized cysteines on diverse mitochondrial proteins.

## 1. Introduction

Mitochondria are the sites of essential cellular metabolic pathways, including the tricarboxylic acid (TCA) cycle and the electron transport chain (ETC). Mitochondria comprise distinct membranes, where the outer (OMM) and inner (IMM) mitochondrial membranes envelop the intermembrane space (IMS) [1]. The IMS is critical for the import and folding of mitochondrial proteins, as well as for apoptotic signaling [2,3,4,5]. The innermost compartment, the matrix, hosts the bioenergetic machinery essential for ATP production [6].

In addition to the central role of mitochondria in cellular metabolism, mitochondria are well-characterized hubs of redox signaling. As one of the primary sites of reactive oxygen species (ROS) production in the cell [7], mitochondria balance the ROS production and metabolism to maintain cellular redox homeostasis. ROS production in the mitochondria results from incomplete electron transfer during aerobic respiration, forming superoxide, and ultimately, a variety of different ROS, including hydrogen peroxide [8]. These ROS can regulate the diverse metabolic and signaling pathways in the mitochondria through cysteine oxidation events. In the presence of ROS, cysteines undergo reversible and irreversible oxidative post-translational modifications (oxPTMs) [9] that transiently affect the protein structure, catalytic activity, complex formation, localization, and degradation [10,11,12,13,14]. There are several characterized sites of cysteine oxidation that affect the mitochondrial protein function [15], including C385 on aconitase (ACO2), which regulates the TCA cycle [16,17], C39 on ND3 subunit of complex I (MT-ND3), which tunes the ETC electron flux [18,19], and C253 on uncoupling protein 1 (UCP1), which disrupts the IMM proton gradient [20,21]. These oxidation events underscore the importance of cysteine oxidation in regulating the metabolic flux as a feedback mechanism for maintaining cell homeostasis.

High levels of mtROS are associated with a variety of pathologies, including cancer [22], diabetes [23,24], neurodegeneration [25], and cardiac disease [26]. Therefore, identifying redox-sensitive cysteines within the mitochondria can provide insight into the redox-regulated metabolic and signaling pathways implicated in disease pathogenesis. Redox proteomics approaches facilitate the identification of redox-sensitive cysteines within a complex proteome. One challenge in performing redox proteomic studies on mitochondrial proteins is the low abundance of these proteins relative to the highly abundant cytosolic and nuclear proteins. Mitochondrial proteins account for only 6% of the human proteome [27,28]; therefore, proteomic analyses of whole-cell lysates result in poor coverage of the mitochondrial proteome. The enrichment of mitochondrial proteins through organelle fractionation [29], targeted probes [30,31,32,33,34,35], proximity labeling (BioID [36,37], APEX [38,39,40], and small molecules [41]) significantly improves this mitochondrial protein coverage.

Coupling these mitochondrial enrichment methods with redox proteomic workflows can facilitate the identification of redox-sensitive cysteines within the mitochondria. Several redox proteomic strategies exist that either directly or indirectly monitor cysteine oxidation. Reactive cysteine profiling, using the isotopic tandem orthogonal proteolysis-activity-based protein profiling (isoTOP-ABPP) platform, indirectly identifies redox-sensitive cysteines by monitoring the oxidation-induced losses in cysteine reactivity. Briefly, isoTOP-ABPP applies a thiol-reactive iodoacetamide-alkyne (IA) probe to monitor the decreases in the cysteine reactivity resulting from oxPTMs. In previous work, isoTOP-ABPP has been used to map the sites of cysteine nitrosation within mitochondrial proteins upon mitochondrial enrichment via differential centrifugation [42]. An alternative redox proteomic approach is the use of oxidative isotopically coded affinity tags (OxICAT [43]), which applies the differential isotopic tagging of reduced and oxidized cysteines to determine the percentage of oxidation. Variations of the OxICAT method have been applied to monitor the cysteine oxidation in the mammalian endoplasmic reticulum [44], yeast [45], bacteria [43], and drosophila [46]. Lastly, mitochondria-targeted probes for sulfinic and sulfenic acids have identified these specific oxidation events within mitochondrial proteins [30,31,32,33,34,35].

Here, we combine differential centrifugation to isolate the mitochondria with the isoTOP-ABPP and OxICAT redox proteomic strategies, in order to study the oxidation of mitochondrial cysteines. Specifically, we rank mitochondrial cysteines by their susceptibility to oxidation with hydrogen peroxide and identify the oxidation events that occur upon the inhibition of the ETC using antimycin A (AMA). The redox-sensitive cysteines we identify comprise well-characterized sites of mitochondrial redox regulation, as well as proteins and pathways that have yet to be fully evaluated for their redox sensitivity.

## 2. Materials and Methods

### 2.1. Biological and Chemical Materials

All the reagents were purchased from Sigma-Aldrich (St. Louis, MO, USA) and Fisher Scientific (Waltham, MA, USA), unless otherwise indicated. All the antibodies (Anti-GAPDH (14C10), Anti-ATPIF1 (D6P1Q), Anti-Histone-H3 (D1H2), Anti-CALR (D3E6), and Anti-DYKDDDK (FLAG)) were purchased from Cell Signaling Technology (Danvers, MA, USA). IA-light (IA-L) and IA-heavy (IA-H) were synthesized in-house according to Abo, M. et al. [47]

### 2.2. Mammalian Cell Culture

HEK293T cells were maintained at 37 °C under an atmosphere of 5% CO_2_ in a DMEM medium (Corning, Corning, NY, USA) supplemented with 10% FBS (Biotechne, Minneapolis, MN, USA) and 1% Anti-Anti (Gibco, Waltham, MA, USA).

### 2.3. Isolation of Mitochondria

HEK293T pellets were washed 3 times with a mitochondrial isolation buffer (10 mM Tris-MOPS, 1 mM EDTA/Tris, 200 mM Sucrose, pH 7.4, IBC). Crude and pure mitochondria (Mito-C and Mito-P) were obtained following the general protocol of differential centrifugation and isopycnic separation by Frezza, C. et al. [29] and Bak, D. et al. [42].

### 2.4. Western Blot Analysis of GAPDH, CALR, Histone H3 and ATPIF1

A Western blot analysis was performed on 25 µg of whole cell (WC), cytosolic (Cyto), crude mitochondrial (Mito-C) and pure mitochondrial (Mito-P) lysates using rabbit anti-GAPDH (1:1000, TBST and 5% bovine serum albumin (BSA)), rabbit anti-Histone H3 (1:1000, TBST and 5% milk)), rabbit anti-CALR (1:1000, TBST and 5% milk)), or rabbit anti-ATPIF1 (1:1000, TBST and 5% bovine serum albumin (BSA)) primary antibodies, followed by an anti-rabbit IgG HRP conjugate (1:2000).

### 2.5. Treatment of HEK293T Cells with Antimycin A

Confluent HEK293T cells were incubated with either 100 µM of Antimycin A in DMSO or an equivalent volume of DMSO for a total of 1 h at 37 °C.

### 2.6. Mass Spectrometry Sample Preparation

#### 2.6.1. isoTOP-ABPP Analysis

The analysis using isoTOP-ABPP was performed according to Weerapana, E. et al. [48], Bak, D. et al. [42], and Abo, M. et al. [47]. For the hydrogen peroxide treatments, 0.5 mg of isolated Mito-P lysates in 500 µL were pretreated in the dark with 1 µL of 100× stocks of hydrogen peroxide or water on ice, for 20 min prior to the IA labeling. For the Antimycin A studies, the IA labeling was performed on 0.5 mg of intact Mito-P fractions prior to lysis.

#### 2.6.2. OxICAT Studies and Cysteine Oxidation Analysis

The OxICAT studies were performed with 4 mg of isolated Mito-P using the protocol described in Bechtel, T. et al. [44].

### 2.7. Tandem MS Analysis

An MS analysis was performed on a Thermo Fisher LTQ Orbitrap Discovery mass spectrometer coupled with an Agilent 1200 series HPLC, as previously described [48].

### 2.8. LC/LC-MS/MS Data Processing

The MS/MS data were analyzed using the SEQUEST algorithm, filtered using DTASelect 2.0 [49,50], and the light:heavy ratios were obtained using CIMAGE [48], as previously described [48]. A dynamic modification of the cysteine for the IA-L (306.14806 *m*/*z*) and IA-H (312.16819 *m*/*z*) adducts was included.

#### 2.8.1. MS Data Analysis: isoTOP-ABPP for Peroxide Treatment

Two replicates of each peroxide concentration (1, 2.5, 5, and 10 mM of H_2_O_2_) were analyzed. The average ratios were calculated for the peptides with ratios present in both replicates, and values exhibiting > 2-fold changes and coefficients of variation of >50% were removed. The data were filtered via mitochondrial localization through a comparison with the Uniprot [51] and MitoCarta3.0 [52] databases.

#### 2.8.2. MS Data Analysis: isoTOP-ABPP for AMA Treatment

Three replicates were analyzed. L:H Ratios were required to be in 2 out of 3 of the replicates. The average ratios of a >2-fold change were filtered by a coefficient of variation cut-off of 50%. Only peptides from the mitochondria-annotated proteins were included.

#### 2.8.3. MS Data Analysis: OxICAT

Three replicates were analyzed to obtain the L:H and H:L ratios. The data were filtered to include only the mitochondrial proteins and peptides that appeared in two out of the three replicates. The L:H and H:L ratios were converted to % oxidation values by using the equations: 1 − (L:H/(L:H + 1)) or H:L/(H:L + 1). The average % oxidation values were determined for each peptide and those with a standard deviation of mean of >30% were removed.

### 2.9. Statistical Analysis of Overrepresented GO Biological Processes Using Panther

Protein ID lists from the whole cell cysteine reactivity data from Weerapana, E. et al. [48] and the Mito-P cysteine reactivity data from the 1 mM peroxide studies were analyzed using Panther 16.0 overrepresentation tests (http:/pantherdb.org (accessed on 21 May 2021)) [53].

## 3. Results

### 3.1. Differential Centrifugation to Increase Coverage of Mitochondrial Cysteines

To enrich the intact mitochondria, we adapted the established methods of differential centrifugation [29,42] to fractionate the HEK293T cell lysates to generate a crude mitochondrial sample (Mito-C) (Figure S1A). The Mito-C fraction was further purified with an isopycnic percoll gradient to produce a pure mitochondrial sample (Mito-P), with minimal cytosolic and nuclear contamination (Figure S1B). An analysis by Western blot using antibodies against the nuclear (histone H3), ER (calreticulin), cytosolic (GAPDH), and mitochondrial (ATPIF1) proteins demonstrated the successful enrichment of the mitochondrial proteins and a loss of the nuclear and cytosolic proteins in the Mito-C and Mito-P fractions. The ER marker, calreticulin, was still present in the Mito-P sample, likely due to the inability of differential centrifugation to disrupt ER–mitochondrial contact sites.

The enrichment of the mitochondrial proteins in the Mito-P fraction was further confirmed by enriching and identifying the reactive cysteines using mass spectrometry (MS) [50]. Briefly, the Mito-P lysates were treated with an iodoacetamide-alkyne (IA) probe to covalently modify their reactive cysteines. The IA-modified proteins were conjugated to a chemically cleavable biotin linker (Figure S2B) using a copper (I)-catalyzed azide-alkyne cycloaddition (CuAAC). Biotinylated proteins were enriched on streptavidin beads, subjected to on-bead trypsin digestion, and there was a subsequent release of the IA-modified peptides using a sodium dithionite treatment. The resulting IA-modified peptides were analyzed with tandem liquid chromatography–mass spectrometry (LC/LC-MS/MS). The mitochondrial cysteine coverage in the Mito-P sample was determined to be similar to that in previous fractionation reports [42], with a total of 1563 cysteines identified from the 481 proteins that were identified to localize to the mitochondria by Uniprot [51] and MitoCarta2.0 [54] (Table S1). Previously reported reactive cysteine profiling studies on unfractionated whole-cell lysates [48] have identified 278 cysteines from 172 mitochondrial proteins. Therefore, mitochondrial isolation resulted in a ~three-fold increase in the mitochondrial proteins and ~five-fold increase in the mitochondrial peptides identified (Figure S1D). Additionally, 57% of the spectral counts in the Mito-P sample originated from the mitochondrial proteins, compared to 11% in the whole-cell sample (Figure S1C). A gene ontology (GO) analysis of the Mito-P sample revealed a robust enrichment for common mitochondrial processes such as mitochondrial protein translation, mitochondrial gene expression, and the TCA cycle, none of which were enriched in the whole-cell sample (Tables S2 and S3, Figure S1E). Together, our data confirm differential centrifugation to be a valuable method for mitochondrial proteome enrichment.

### 3.2. Monitoring the Redox Sensitivity of Mitochondrial Cysteines

**Figure 1:**
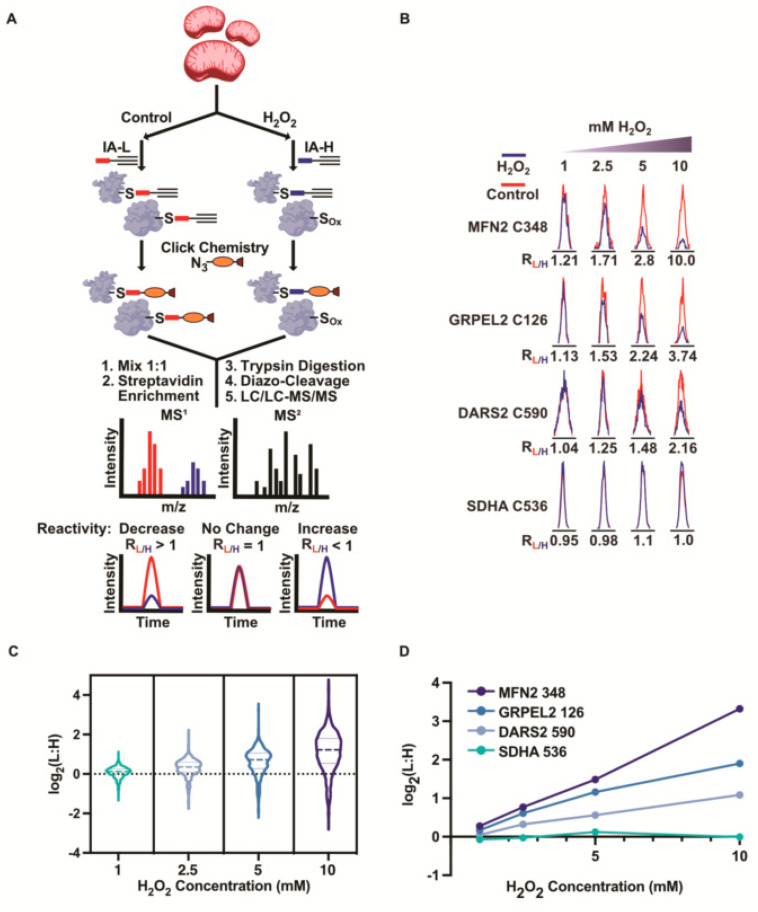
Analysis of mitochondrial cysteine reactivity in the presence of peroxide. (**A**) Workflow for evaluating peroxide-dependent changes in cysteine reactivity in isolated mitochondrial lysates using isoTOP-ABPP. Cysteines from control or peroxide-treated Mito-P lysates are labeled with IA-L or IA-H, respectively. Cleavable biotin-azide tags are then appended followed by enrichment of labeled cysteine-containing proteins on streptavidin resin, trypsin digestion, and isolation of labeled cysteine-containing peptides for quantitative MS analysis to identify and quantify cysteine reactivity changes due to oxidation by peroxide. (**B**) Violin plot displaying median L:H ratios for all identified cysteines with increasing concentrations of peroxide (1 mM, 2.5 mM, 5 mM, and 10 mM H_2_O_2_). (**C**) Plotted average L:H ratios (log_2_L:H) of mitochondrial cysteines with increasing peroxide treatments. (**D**) Representative extracted ion chromatograms of cysteines from (**C**) alkylated by IA-L (red) or IA-H (blue) in the control and peroxide-treated samples, respectively.

Upon confirming this mitochondrial enrichment, we applied the isoTOP-ABPP platform to rank the mitochondrial cysteines by their sensitivity to hydrogen peroxide (Figure 1A) [47,48]. IsoTOP-ABPP compared the cysteine reactivity across two biological samples through the application of isotopically labeled IA probes, IA-L (light), and IA-H (heavy) (Figure S2A). The Mito-P fractions were treated with 1, 2.5, 5, and 10 mM of hydrogen peroxide for 20 min. These peroxide-treated samples were then labeled with IA-H, and a corresponding untreated control sample was labeled with IA-L. The peroxide-treated and untreated samples were mixed together prior to the streptavidin enrichment, trypsin digestion, and sodium dithionite elution. The resulting peptide mixtures were analyzed using LC/LC-MS/MS. For every cysteine-containing peptide that was identified, the relative IA-labeling in the untreated controls versus the peroxide-treated experimental samples could be determined by the light:heavy (L:H) ratios. An L:H ratio of >1 corresponded to a decrease in the cysteine reactivity upon the peroxide treatment, and was indicative of cysteine oxidation. An L:H ratio equivalent to 1 indicated an unchanged cysteine reactivity upon the peroxide treatment. Importantly, the use of this isoTOP-ABPP analysis allowed for a determination of the extent of oxidation, whereby the higher the L:H ratio value, the greater the stoichiometry of the oxidation. The peroxide concentrations used were supraphysiological, but were selected such that minimal oxidation was observed at the lowest concentration (1 mM) and a high-stoichiometry oxidation for a subset of cysteines was present at the highest peroxide concentration (10 mM). Importantly, we ensured that, at the highest (10 mM) concentration of peroxide, we did not see the complete oxidation of all the cysteines, indicating that we were not completely overwhelming the oxidation capacity of the system.

The isoTOP-ABPP analysis identified ~700 mitochondrial cysteines (Tables S4–S7) with robust L:H ratios in each of the peroxide treatments. As expected, increasing median L:H ratios were observed with increasing peroxide concentrations (Figure 1B), indicating proteome-wide decreases in cysteine reactivity. For example, a 1 mM peroxide treatment led to very minor changes in cysteine reactivity (a median L:H ratio of 1.1). In contrast, the 10 mM peroxide treatments displayed pronounced decreases in cysteine reactivity (a median L:H ratio of 2.3). The treatments of 2.5 mM and 5 mM peroxide gave median L:H ratios of 1.3 and 1.7, respectively.

Importantly, the effect of the peroxide was not uniform across the identified cysteines, allowing us to group the cysteines within groupings of a low, moderate, and high sensitivity to the peroxide treatments. A subset of cysteines displayed no change in their cysteine reactivity (L:H ratios ~1), regardless of the peroxide concentration, indicating a resistance to oxidation with hydrogen peroxide. These cysteines included C536 on succinate dehydrogenase A (SDHA) (Figure 1C). The cysteines that were oxidation-sensitive displayed variations in their concentration dependence, underscoring the unique redox susceptibilities of each individual cysteine. Some cysteines, such as C348 on Mitofusin 2 (MFN2), displayed steep linear decreases in their reactivity, indicating a high susceptibility to oxidation. MFN2 is an OMM protein central to mitochondrial network remodeling and inter-organelle contact (Figure 1C). MFN2 dysfunction has been implicated in mitophagy, unfolded protein responses, and the metabolic dysregulation characteristics of neurodegeneration, cardiomyopathy, and cancer [55]. C348 has not been previously identified to be redox-sensitive, but C684 is known to form an intermolecular disulfide with a cysteine on MFN1 to promote mitochondrial fusion [56]. Notably, C348 on MFN2 is located prior to an HR1 (heptad-repeating coiled-coil) domain analogous to the positioning of C684 and the HR2 domain, thereby supporting a potential redox function for C348, similar to C684 [57]. The cysteines that displayed a moderate peroxide sensitivity included C126 on the matrix-residing GrpE protein homolog 2 (GRPEL2) and C590 on mitochondrial aspartate tRNA ligase (DARS2) (Figure 1C). The trends in the cysteine redox sensitivity were further visualized with extracted ion chromatograms for each IA-L- and IA-H-labeled peptide (Figure 1D). Together, these concentration-dependent analyses enabled the ranking of the mitochondrial cysteines by their sensitivity to peroxide-mediated oxidation.

### 3.3. Cysteine-Reactivity Changes of Known Redox-Sensitive Proteins

**Figure 2:**
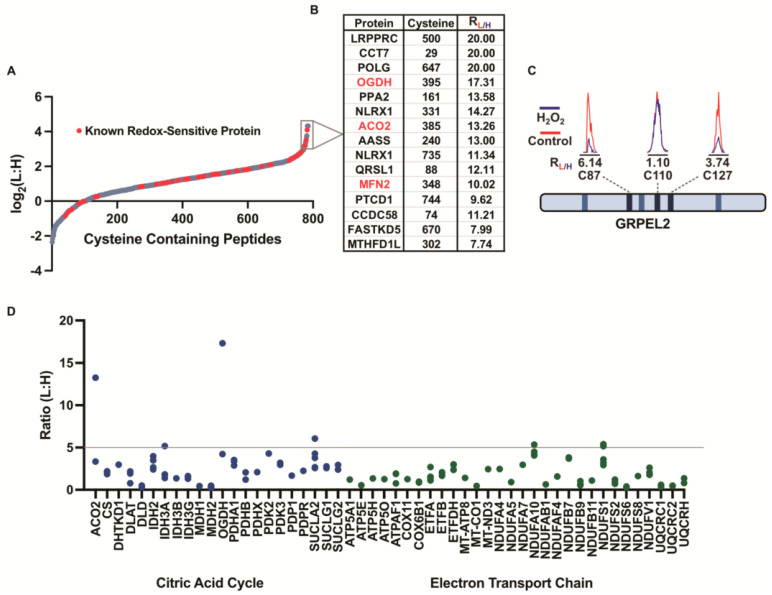
Evaluation of cysteine reactivity changes under 10 mM peroxide treatment using IsoTOP-ABPP. (**A**) L:H ratio plot (log_2_L:H) of mitochondrial cysteines identified by isoTOP-ABPP upon 10 mM peroxide treatment. Previously characterized redox-sensitive proteins are annotated in red. (**B**) Inset highlights cysteine-containing peptides with the highest L:H ratios and previously characterized redox-sensitive proteins are in red. (**C**) Representative extracted ion chromatograms for cysteines identified from GRPEL2. Blue traces are from the untreated control and red traces are from the peroxide-treated sample. (**D**) Average L:H ratios for cysteines identified on proteins belonging to mitochondrial bioenergetic pathways including the citric acid cycle (blue), urea cycle (red), and electron transport chain (green) in the 10 mM peroxide isoTOP-ABPP dataset. High peroxide sensitivity (L:H ratio of 5) is denoted by a gray line.

We focused on the dataset for the Mito-P lysates that were exposed to 10 mM of the peroxide (Figure 2A, Table S7), where the largest extent of cysteine oxidation was observed. Of the 785 cysteines that generated robust L:H ratios, 454 (58%) revealed at least a two-fold decrease in their reactivity, including 65 (8%) cysteines that displayed a higher than five-fold decrease. Within these highly peroxide-sensitive cysteines were well-characterized sites of oxidation, including C385 on aconitase 2 (ACO2) (an L:H ratio of 13.3) and C395 on 2-oxoglutarate dehydrogenase (OGDH) (an L:H ratio of 17.3) (Figure 2B). The oxidation of ACO2 and OGDH is known to inhibit protein function and attenuate the TCA cycle [17,58]. Interestingly, OGDH catalyzes the rate-limiting step of the TCA cycle and is the highest generator of mtROS outside of the ETC. OGDH is also known to be self-regulated through cysteine modifications such as glutathionylation [59,60]. The precise site(s) of cysteine oxidation that result in OGDH inhibition remain uncharacterized, however, C395 on OGDH has been previously shown to undergo *S*-nitrosation in a biotin-switch study on cardiac mitochondria [60].

Our data confirm that cysteines within a single protein can display widely divergent redox sensitivities. For instance, we identified three cysteines on GRPEL2 with varying redox sensitivities (Figure 2C). In the presence of the peroxide, the reactivity of C110 was preserved (an L:H ratio of 1.1), while C87 and C127 displayed L:H ratios of 6.1 and 3.7, respectively. The most redox-sensitive cysteine, C87, is known to facilitate a GRPEL2 dimer formation that protects against proteolysis during oxidative stress [61]. The identification of well-characterized sites of cysteine oxidation serves to validate the ability of our platform to accurately report on the redox sensitivities of mitochondrial cysteines.

### 3.4. Levels of Cysteine Oxidation within Key Mitochondrial Pathways

The most well-characterized redox-sensitive cysteines are known to reside within known ROS-regulated pathways [15]. Therefore, we sought to explore if the highly redox-sensitive cysteines that we identified were concentrated within specific mitochondrial processes or pathways. The cysteines identified from the 10 mM peroxide dataset were binned into 12 well-established biochemical classifications: the TCA cycle, innate immunity, transport, the urea cycle, fatty acid oxidation, redox regulation, Fe-S biogenesis, apoptosis, tRNA-ligases, mitochondrial (mt) ribosomal subunits, ETC, and mitochondrial transcription and translation (Figure 2D andFigure S4). We observed that the redox-sensitive cysteines were distributed across these pathways and processes, but that some pathways had an increased number of highly redox-sensitive cysteines (>five-fold decrease in reactivity). Within the known redox-regulated metabolic pathways, the TCA cycle contained four proteins with highly redox-sensitive cysteines. These included the characterized redox-sensitive C385 on ACO2 and C395 on OGDH, which were mentioned previously, as well as poorly characterized cysteines on isocitrate dehydrogenase, IDH3A, and succinyl-CoA ligase, SUCLA2. Several subunits of the ETC (NDUFA10 and NDUFS1) also contained cysteines with a high redox sensitivity. Interestingly, the proteins involved in mitochondrial transcription and translation contained a high abundance of these redox-sensitive cysteines, including subunits of the mitochondrial ribosome, mRNA-processing enzymes, and DNA polymerase subunits. In support of this observation, the recent literature indicates that mtROS can regulate mitochondrial ribosome and aminoacyl tRNA complex formation [45,62]. Together, our data identify the highly redox-sensitive cysteines within the pathways that are known to be redox-regulated.

### 3.5. Inhibition of the Electron Transport Chain Induces ROS Generation and Cysteine Oxidation

**Figure 3:**
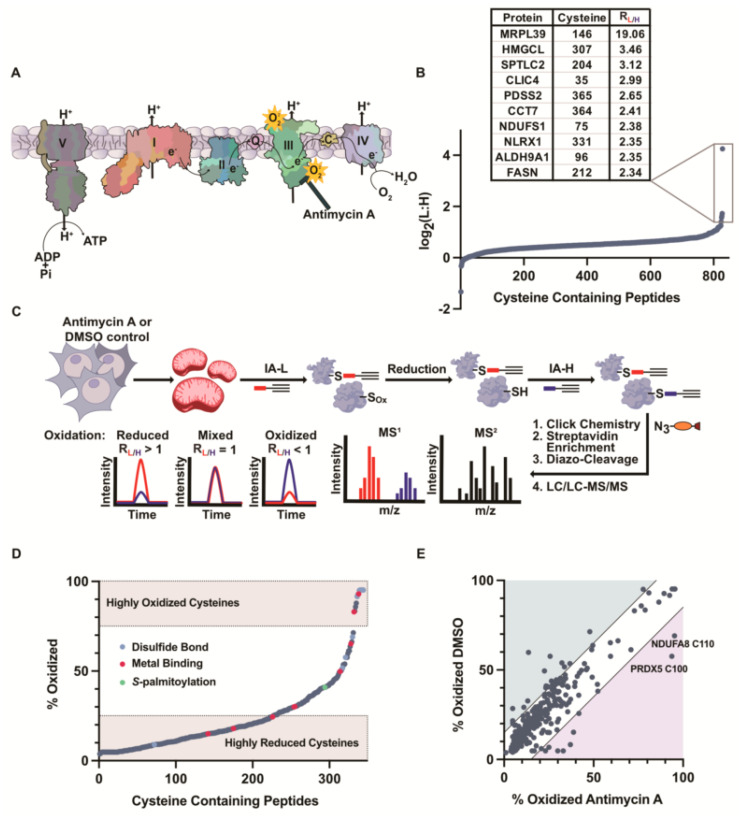
Cysteine reactivity and oxidation profiling under ETC inhibition. (**A**) Depiction of ETC inhibition of complex III by Antimycin A (AMA). Electron leakage and superoxide production occurs in both the mitochondrial matrix and IMS. (**B**) L:H ratio plot (log_2_L:H) of mitochondrial cysteine-containing peptides identified in isoTOP-ABPP analysis of Mito-P from AMA-treated cells. (**C**) OxICAT workflow in isolated mitochondria from DMSO-treated cells (control) or AMA-treated cells. Workflow includes differential alkylation of reduced and oxidized cysteine thiols with IA-L or IA-H followed by incorporation of cleavable biotin-azide tag for labeled cysteine-containing protein enrichment, trypsin digestion, and labeled cysteine-containing peptide isolation. L:H ratios directly correspond to percent oxidation values of cysteines. (**D**) Percent oxidation values for DMSO-treated mitochondria (control). UniProt annotation of cysteine residues that are disulfide linked (blue), metal binding (red) and s-palmitoylation (green). Percent oxidation is directly calculated from L:H and H:L ratios. (**E**) Percent oxidation values for DMSO- and AMA-treated mitochondria.

The hydrogen peroxide treatments were geared toward enabling the ranking of the mitochondrial cysteines by their relative susceptibility to oxidation. The concentrations of peroxide that were used were supraphysiological, so as to push the levels of oxidation to a high stoichiometry that would differentiate the cysteines with high, medium, and low sensitivities to oxidation. The highly redox-sensitive cysteines that we identified correlated with the cysteines that were previously shown to be oxidized within (patho)physiological systems, suggesting that a high redox susceptibility in vitro can be predictive of in vivo sensitivity. To further evaluate this cysteine oxidation under physiological ROS conditions, we applied isoTOP-ABPP to assess the changes in cysteine reactivity and oxidation upon the inhibition of complex III with Antimycin A (AMA) (Figure 3A) [63], in order to release mtROS into the matrix and IMS.

To investigate the cysteine oxidation upon ETC inhibition, the cells were incubated with either AMA or DMSO (control) for 1 h prior to the fractionation and Mito-P isolation. The intact Mito-P fractions from the AMA-treated cells were labeled with IA-H, while the Mito-P from the control cells was labeled with IA-L. After lysis, the isoTOP-ABPP workflow was followed as previously described (Figure S3). The AMA-induced oxidation of a cysteine resulted in an increased L:H ratio. The resulting MS analysis provided robust L:H ratios for 828 cysteines on 432 mitochondrial proteins (Table S8). The proteome-wide impact of AMA on the cysteine reactivity was much less severe than that with an exogenous 10 mM peroxide treatment, generating a median L:H ratio of 1.4 compared to 2.3 for the peroxide.

The majority of the cysteines displayed L:H ratios of ~1 and were not affected by AMA. However, a small subset of 31 cysteines displayed >two-fold decreases in their reactivity, signifying increased oxidation upon the AMA treatment. Interestingly, C146 on Mitochondrial Ribosomal Protein L39 (MRPL39) exhibited the highest L:H value of 19.1 in the AMA-treated dataset. Based on the available structural data, C146 on MRPL39 is located proximal to C275 and could potentially form a disulfide bond under oxidative stress. Although MRPL39 has not been shown to be redox-regulated, proximal cysteines on adjacent MRPs have been identified to form disulfide bonds [64]. Furthermore, in yeast OxICAT studies [45], numerous proteins involved in mitochondrial translation have demonstrated high oxidation in the presence of peroxide, including MRPL32, indicating the potential for the MRP family, in general, to be regulated by mtROS [45,65].

The correlation of the peroxide and AMA datasets identified cysteines that displayed decreases in their reactivity under both conditions. For instance, C331 on NLR Family Member X1 (NLRX1) displayed an average L:H ratio of 14.3 with 10 mM peroxide and 2.4 with AMA. NLRX1 is involved in antiviral signaling and injury prevention via the potentiation of ROS-driven immune activation [66]. NLRX1 is also known to negatively regulate the mitochondrial anti-viral signaling protein (MAVS), which is a redox-regulated protein that undergoes ROS-dependent oligomerization. Despite the implication of NLRX1 in ROS-dependent pathways, there has been no report of NLRX1 cysteines being the target of oxidation. Our studies identify C331 as a potential site of oxidation on NLRX1.

### 3.6. Monitoring Mitochondrial Cysteine Oxidation with OxICAT

Cysteine reactivity changes, as measured by isoTOP-ABPP, provide an indirect measure of cysteine oxidation through the observed decreases in the cysteine reactivity. A more direct method for analyzing this cysteine oxidation is through OxICAT [39], which uses the differential isotopic tagging of reduced and oxidized cysteines to directly monitor the cysteine oxidation within a single sample. Briefly, the proteins were denatured and the free thiols were alkylated with IA-L, followed by a reduction in the reversibly oxidized thiols and a subsequent labeling with IA-H (Figure 3C). Upon MS analysis, the resulting L:H ratios could be converted into % oxidation values to report on the stoichiometry of the oxidation for each identified cysteine. The Mito-P isolated from the AMA- or DMSO (control)-treated whole cells were subjected to OxICAT analyses.

In the control cells (Figure 3D, Table S9), a small subset of cysteines (8%) were found to be over 50% oxidized and included several annotated disulfide-linked and metal-binding cysteines. The disulfide-linked cysteines included C50 on mitochondrial import inner membrane translocase subunit TIMM13, which was 95% oxidized. This cysteine lies within a Cx3C motif and is predicted to participate in an intramolecular disulfide bond with C65. Additionally, the active site C80 on dihydrolipoamide dehydrogenase (DLD) was found to be highly oxidized (95%). This cysteine is known to form a redox-active disulfide bond with C85. Lastly, several disulfide-linked cysteines, C30, C54, and C65, on cytochrome C oxidase subunit 6B1 (COX6B1), were identified, and each were 95% oxidized.

AMA treatment results in an observable increase in the oxidation for several cysteines (Figure 3E, Table S9). The cysteines that displayed increased oxidation upon AMA treatment included the peroxidatic cysteine, C100, on peroxiredoxin 5, PRDX5, with a 58% oxidation in the control sample compared to a 94% oxidation in the AMA-treated sample. PRDX5 is an antioxidant enzyme critical in the regulation of peroxide levels and protection of the cell from irreversible oxidative damage under high levels of oxidative stress [67]. C100 is known to become sulfenylated and form a redox-active disulfide bond with C204 [68,69]. Interestingly, the resolving cysteine of PRDX5, C204, was identified to have a 92% oxidation in both the control and AMA-treated samples. Lastly, C110 on NADH dehydrogenase 1 alpha subcomplex subunit (NDUFA8), a complex I subunit, displayed increased oxidation upon the AMA treatment (95% oxidized) compared to the control treatment (70% oxidized). This cysteine is a part of the four CX_9_C motifs on NDUFA8 that undergo a disulfide exchange, which is dependent on the redox conditions of the IMS [70,71]. The identification of the known sites of cysteine oxidation supports the use of mitochondrial fractionation and OxICAT to directly monitor these sites upon the induction of endogenous oxidative stress.

## 4. Discussion

Mitochondria are intricate organelles central to a variety of cellular functions, including aerobic respiration. Many mitochondrial processes can be regulated by an ROS-mediated oxidation of the key mitochondrial cysteines. Although several sites of cysteine oxidation on mitochondrial proteins and the functional consequences of this oxidation have been identified, there is a continued interest in globally assessing the redox sensitivity of these mitochondrial cysteines. Identifying new sites of cysteine oxidation in mitochondria can unearth uncharacterized redox-regulated proteins and pathways.

Here, we describe the use of mitochondrial enrichment by differential centrifugation coupled with redox proteomic platforms, such as isoTOP-ABPP and OxICAT, to identify redox-sensitive cysteines under exogenous and endogenous ROS treatments. The differential centrifugation afforded Mito-P fractions that were analyzed using mass spectrometry and confirmed to be highly enriched in mitochondrial proteins, relative to the whole-cell samples. These Mito-P fractions were then subjected to exogenous hydrogen peroxide treatments and analyzed using isoTOP-ABPP. The hydrogen peroxide concentrations used were supraphysiological, but were chosen to differentiate the cysteines with a high sensitivity to oxidation (highly oxidized at the lowest peroxide concentration used) from those with moderate and low sensitivities to oxidation. Our data enabled the rank ordering of the mitochondrial cysteines by their sensitivity to oxidation and identified the mitochondrial pathways that were enriched in highly redox-sensitive cysteines. Importantly, many of the cysteines identified within the high redox sensitivity category had been previously reported to be oxidized under physiologically relevant levels of oxidative stress.

We then treated cells with the ETC inhibitor Antimycin and applied isoTOP-ABPP and OxICAT to identify the cysteines that were oxidized upon the induction of endogenous reactive oxygen species. The isoTOP-ABPP platform is an indirect method that identifies sites of oxidation through a loss of cysteine reactivity, whereas OxICAT more directly monitors reversible cysteine oxidation events through differential cysteine labeling. These two methods are complementary to each other and differ in their cysteine coverage and the types of oxPTMs that are detectable. Their key differences include the fact that OxICAT will only identify reversible oxidation events (i.e., sulfenic acids and disulfides), whereas isoTOP-ABPP can identify both reversible and irreversible sites of oxidation. Additionally, fully oxidized cysteines, such as those engaged in stable disulfide linkages, can only be identified via an OxICAT analysis, as cysteines that are 100% oxidized are not captured by an isoTOP-ABPP analysis. Lastly, isoTOP-ABPP applies low (100 uM) concentrations of a cysteine-reactive probe to fully folded proteins, resulting in the labeling of only highly reactive cysteines. In contrast, OxICAT applies high (10 mM) concentrations of a cysteine-alkylating agent to fully denatured proteomes, resulting in the labeling of all cysteines, regardless of their reactivity. For these reasons, the cysteines identified by these two methods do not show a complete overlap (Figure S5) and provide access to different subsets of cysteines. The use of both platforms provides a broader snapshot of the cysteine oxidation events that occur in the mitochondria upon the induction of reactive oxygen species. As with the exogenous peroxide treatment, many of the cysteines that were identified to be oxidized in the isoTOP-ABPP and OxICAT analyses of the antimycin-treated mitochondria were previously reported as sites of cysteine oxidation.

## 5. Conclusions

In summary, we combine isoTOP-ABPP and oxICAT redox proteomic methods, together with mitochondrial isolation, to provide a snapshot of the cysteine oxidation events that accompany both exogenous and endogenous ROS exposure. Our combined datasets identify many previously characterized sites of oxidation, but also unearth unannotated sites of oxidation on proteins that could potentially regulate diverse mitochondrial processes under oxidative stress. This work sets the foundation for a more extensive exploration of mitochondrial cysteine oxidation. The use of more stringent mitochondrial fractionation methods, as well as an improved MS sensitivity, will likely serve to improve mitochondrial cysteine coverage.

## Data Availability

All data in the manuscript are available from the corresponding author.

## Supplementary Materials

The following supporting information can be downloaded at: https://www.mdpi.com/article/10.3390/antiox12050992/s1, Figure S1: Evaluation of mitochondrial isolation by differential centrifugation; Figure S2: Probe structures used for MS experiments; Figure S3: Workflow for quantifying redox-dependent changes in cysteine reactivity in isolated mitochondria using IsoTOP-ABPP; Figure S4: L:H ratios plotted from isoTOP-ABPP analysis of 10 mM peroxide treated Mito-P samples; Figure S5: Overlap between isoTOP-ABPP analysis and OxICAT analysis for all identified cysteines; Table S1: Annotated list of mitochondrial peptides identified in the Mito-P sample; Table S2: Gene ontology (GO) analysis for enrichment of biological processes for proteins identified in whole cell sample; Table S3: Gene ontology (GO) analysis for enrichment of biological processes for proteins identified in whole cell sample; Table S4: Filtered list of L:H ratio values obtained for isoTOP-ABPP analysis of all mitochondrial cysteines identified in Mito-P lysates treated with 1 mM H_2_O_2_; Table S5: Filtered list of L:H ratio values obtained for isoTOP-ABPP analysis of all mitochondrial cysteines identified in Mito-P lysates treated with 2.5 mM H_2_O_2_; Table S6: Filtered list of L:H ratio values obtained for isoTOP-ABPP analysis of all mitochondrial cysteines identified in Mito-P lysates treated with 5 mM H_2_O_2_; Table S7: Filtered list of L:H ratio values obtained for isoTOP-ABPP analysis of all mitochondrial cysteines identified in Mito-P lysates treated with 10 mM H_2_O_2_; Table S8: Filtered list of L:H ratio values obtained from IsoTOP-ABPP analysis of all mitochondrial cysteines identified in Mito-P isolated from whole cells treated with Antimycin A (AMA) or DMSO for 1 h; Table S9: Filtered list of L:H ratios and % oxidation values calculated for mitochondrial cysteines identified in OxICAT analysis of Mito-P isolated from whole cells treated with Antimycin A (AMA) or DMSO for 1 h.
