# Supplementary material for: Identifying Redox-Sensitive Cysteine Residues in Mitochondria
Source: Antioxidants (Basel). 2023 Apr 25;12(5):992. doi: 10.3390/antiox12050992 (PMC10215197; doi:10.3390/antiox12050992)
Supplement: Supplementary file 1 [file antioxidants-12-00992-s001.zip › antioxidants-2289424-supplementary/Supplemetary Documents/antioxidants-2289424-supplementary.pdf]

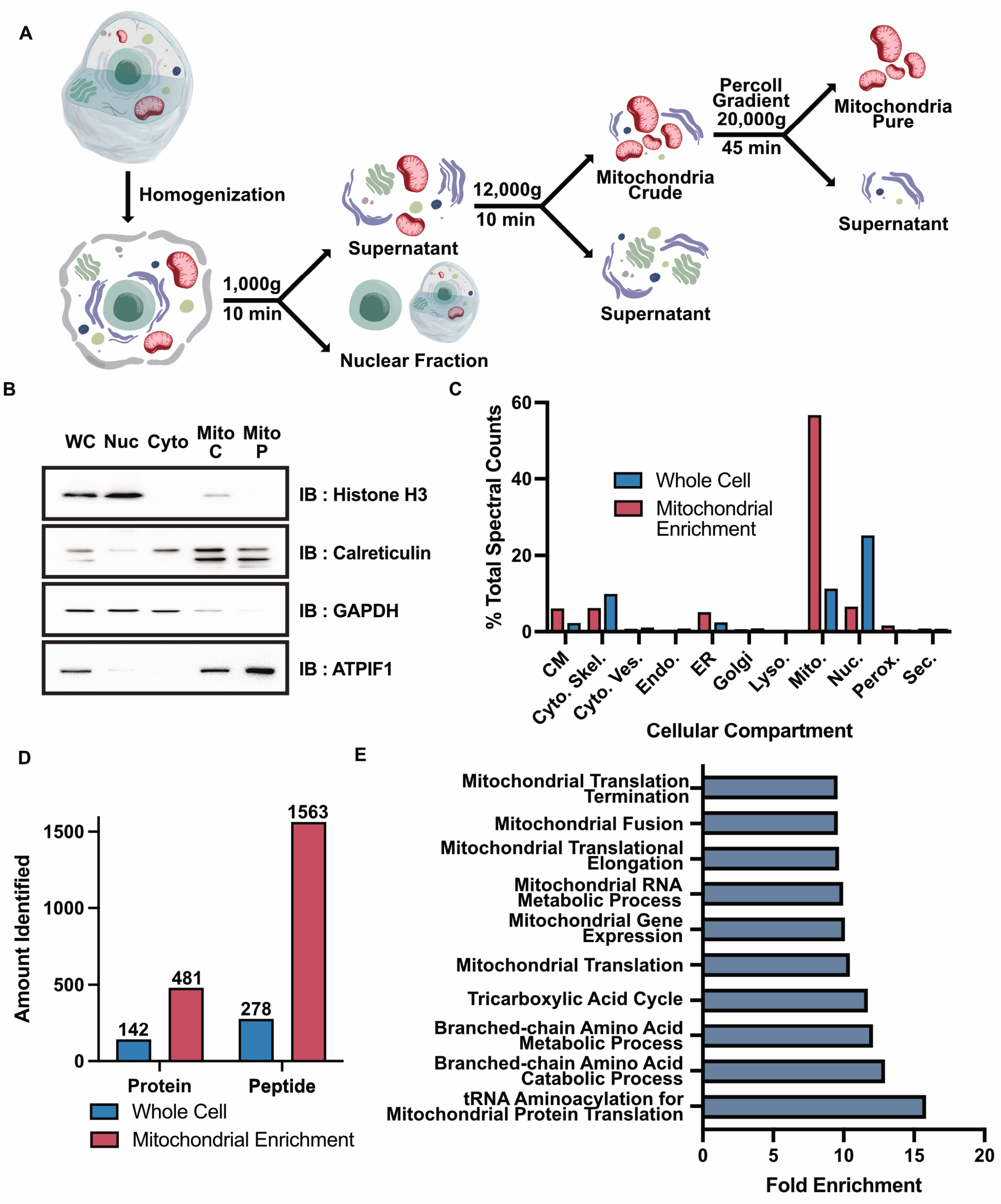

A
Homogenization
1,000g
10 min
Supernatant
Nuclear Fraction
12,000g
10 min
Mitochondria Crude
Supernatant
Percoll Gradient 20,000g
45 min
Mitochondria Pure
Supernatant
B
WC Nuc Cyto Mito C Mito P
IB : Histone H3
IB : Calreticulin
IB : GAPDH
IB : ATPIF1
C
% Total Spectral Counts
Whole Cell
Mitochondrial Enrichment
CM Cyto. Skel. Cyto. Ves. Endo. ER Golgi Lyso. Mito. Nuc. Perox. Sec.
Cellular Compartment
D
Amount Identified
142 481 278 1563
Protein Peptide
Whole Cell
Mitochondrial Enrichment
E
Mitochondrial Translation Termination
Mitochondrial Fusion
Mitochondrial Translational Elongation
Mitochondrial RNA Metabolic Process
Mitochondrial Gene Expression
Mitochondrial Translation
Tricarboxylic Acid Cycle
Branched-chain Amino Acid Metabolic Process
Branched-chain Amino Acid Catabolic Process
tRNA Aminoacylation for Mitochondrial Protein Translation
Fold Enrichment

Figure S1. Evaluation of mitochondrial isolation by differential centrifugation

(A) Differential centrifugation workflow for mitochondrial isolation from whole cells.

(B) Western blot analysis of isolated subcellular fractions during mitochondrial enrichment by differential centrifugation using organelle markers including Histone H3 (nuclear), Calreticulin (endoplasmic reticulum), GAPDH (cytosol), and ATPIF1 (mitochondria). "WC" depicts the whole cell fraction, "Nuc" depicts the nuclear fraction, and "Cyto" depicts the cytosolic fraction. "Mito-C" depicts the crude mitochondrial fraction and "Mito-P" depicts the pure mitochondrial fraction.

(C) Bar graph of the percentage of total spectral counts belonging to proteins with specified UniProt sub-cellular localizations in a whole cell (blue) and isolated mitochondria (red) samples. Annotated compartments include cell membrane (CM), cytoskeleton (Cyto. Skel.), endosome (Endo.), endoplasmic reticulum (ER), golgi apparatus (Golgi), lysosome (Lyso.), mitochondria (Mito.), nucleus (Nuc.), peroxisome (Perox.), and secreted (Sec.).

(D) Bar graph of the total annotated mitochondrial proteins and peptides identified from whole cell (blue) or isolated mitochondria (red).

(E) Gene ontology analysis of the enriched biological processes in the Mito-P sample.

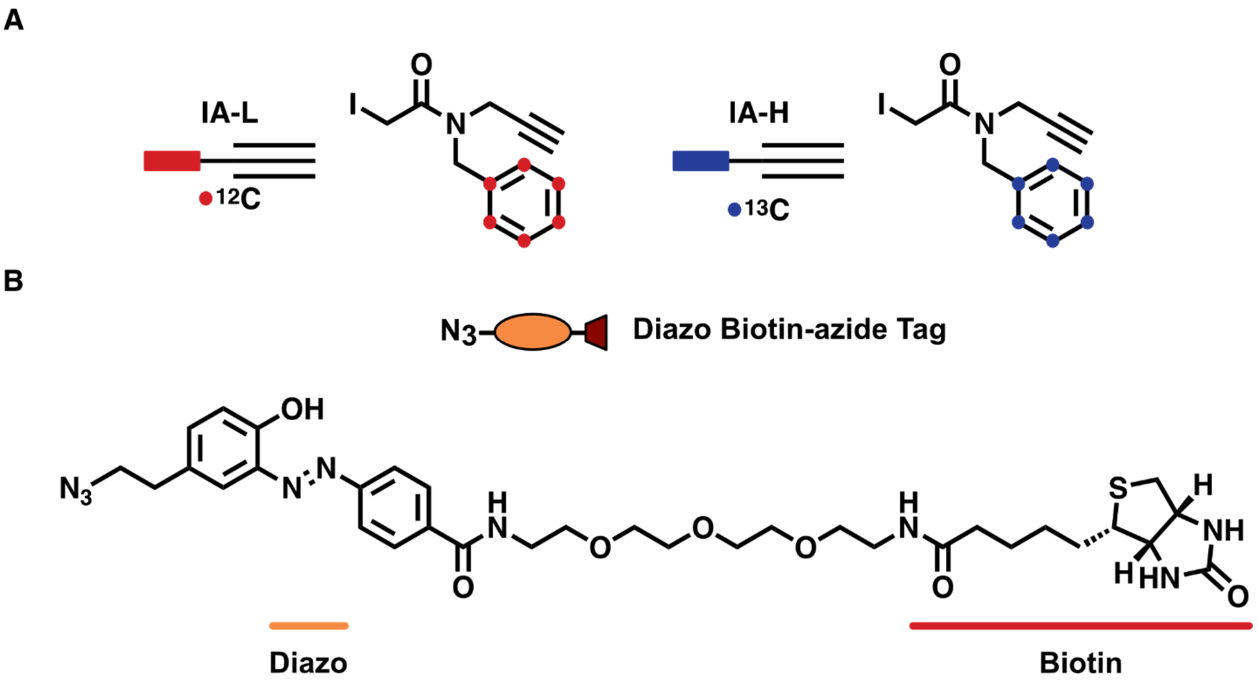

Figure S2. Probe structures used for MS experiments.

(A) Isotopically coded alkylating reagents, light (IA-L, ^{12}C) and heavy (IA-H, ^{13}C) iodoacetamide alkyne.

(B) Structure of cleavable diazo biotin-azide tag.

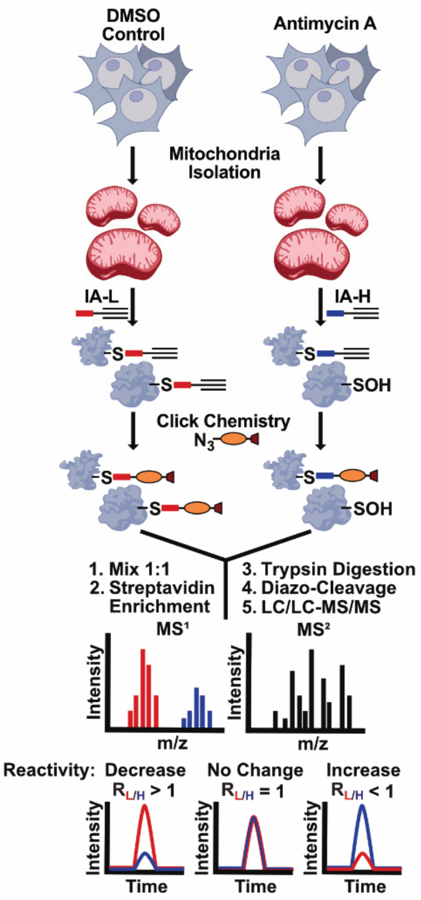

Figure S3. Workflow for quantifying redox-dependent changes in cysteine reactivity in isolated mitochondria using IsoTOP-ABPP. Labeling of thiols from DMSO control or Antimycin A (AMA) treated samples with IA-L or IA- H, respectively, appending cleavable biotin-azide tags, enrichment of labeled cysteine containing proteins, trypsin digestion, and isolation of labeled cysteine-containing peptides for quantitative MS to identify and quantify cysteine reactivity changes due to oxidation.

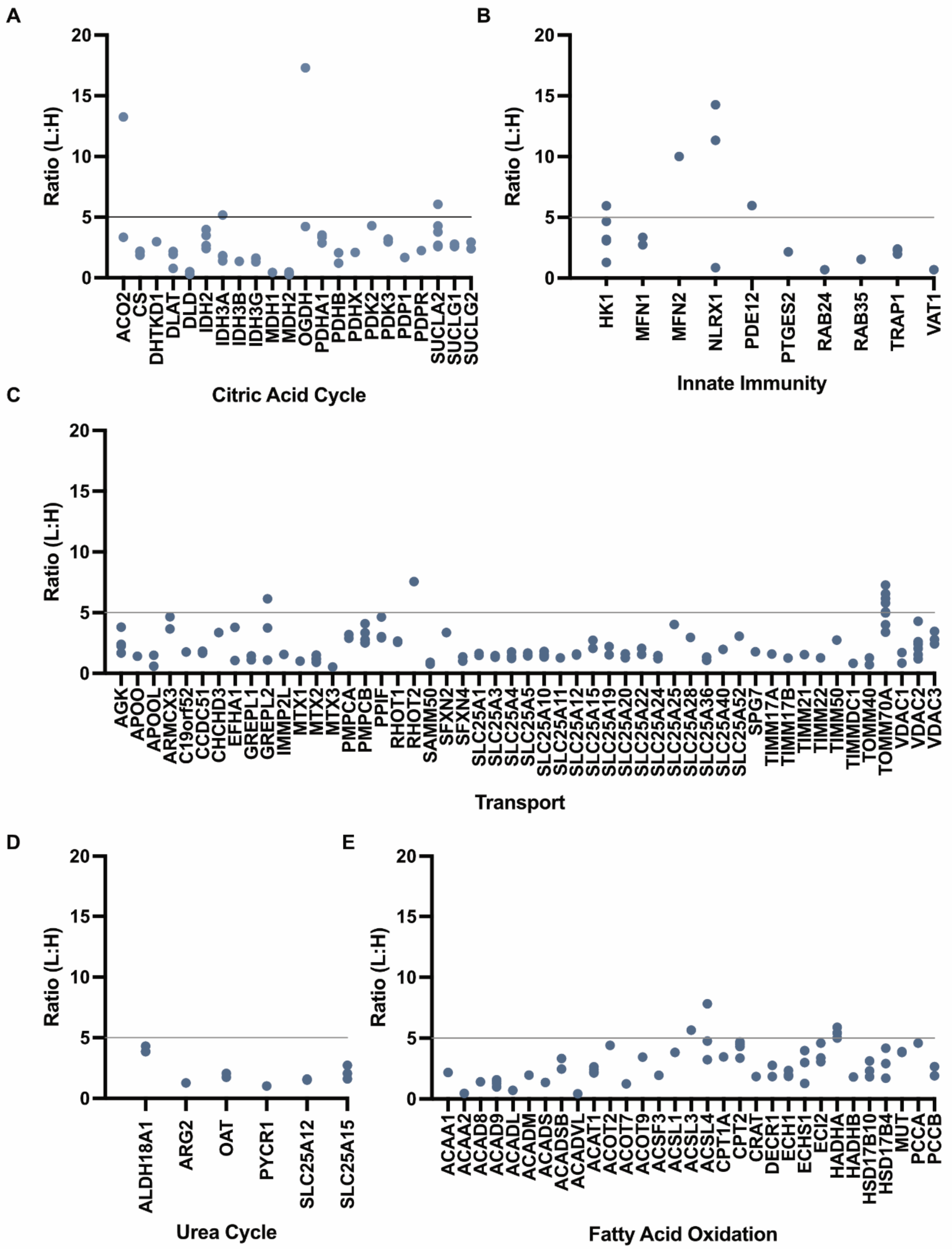
A
Ratio (L:H)
20
15
10
5
0
ACO2
CS
DHTKD1
DLAT
DLD
IDH2
IDH3A
IDH3B
IDH3G
MDH1
MDH2
OGDH
PDHA1
PDHB
PDHX
PDK2
PDK3
PDP1
PDPR
SUCLA2
SUCLG1
SUCLG2
Citric Acid Cycle
B
Ratio (L:H)
20
15
10
5
0
HK1
MFN1
MFN2
NLRX1
PDE12
PTGES2
RAB24
RAB35
TRAP1
VAT1
Innate Immunity
C
Ratio (L:H)
20
15
10
5
0
AGK
APOO
APOOL
ARMCX3
C19orf52
CCDC51
CHCHD3
EFHA1
GREPL1
GREPL2
IMMP2L
MTX1
MTX2
MTX3
PMPCA
PMPCB
PPIF
RHOT1
RHOT2
SAMM50
SFXN2
SFXN4
SLC25A1
SLC25A3
SLC25A4
SLC25A5
SLC25A10
SLC25A11
SLC25A12
SLC25A15
SLC25A19
SLC25A20
SLC25A22
SLC25A24
SLC25A25
SLC25A28
SLC25A36
SLC25A40
SLC25A52
SPG7
TIMM17A
TIMM17B
TIMM21
TIMM22
TIMM50
TIMMDC1
TOMM40
TOMM70A
VDAC1
VDAC2
VDAC3
Transport
D
Ratio (L:H)
20
15
10
5
0
ALDH18A1
ARG2
OAT
PYCR1
SLC25A12
SLC25A15
Urea Cycle
E
Ratio (L:H)
20
15
10
5
0
ACAA1
ACAA2
ACAD8
ACAD9
ACADL
ACADM
ACADS
ACADSB
ACADVL
ACAT1
ACOT2
ACOT7
ACOT9
ACSF3
ACSL1
ACSL3
ACSL4
CPT1A
CPT2
CRAT
DECR1
ECH1
ECHS1
ECI2
HADHA
HADHB
HSD17B10
HSD17B4
MUT
PCCA
PCCB
Fatty Acid Oxidation

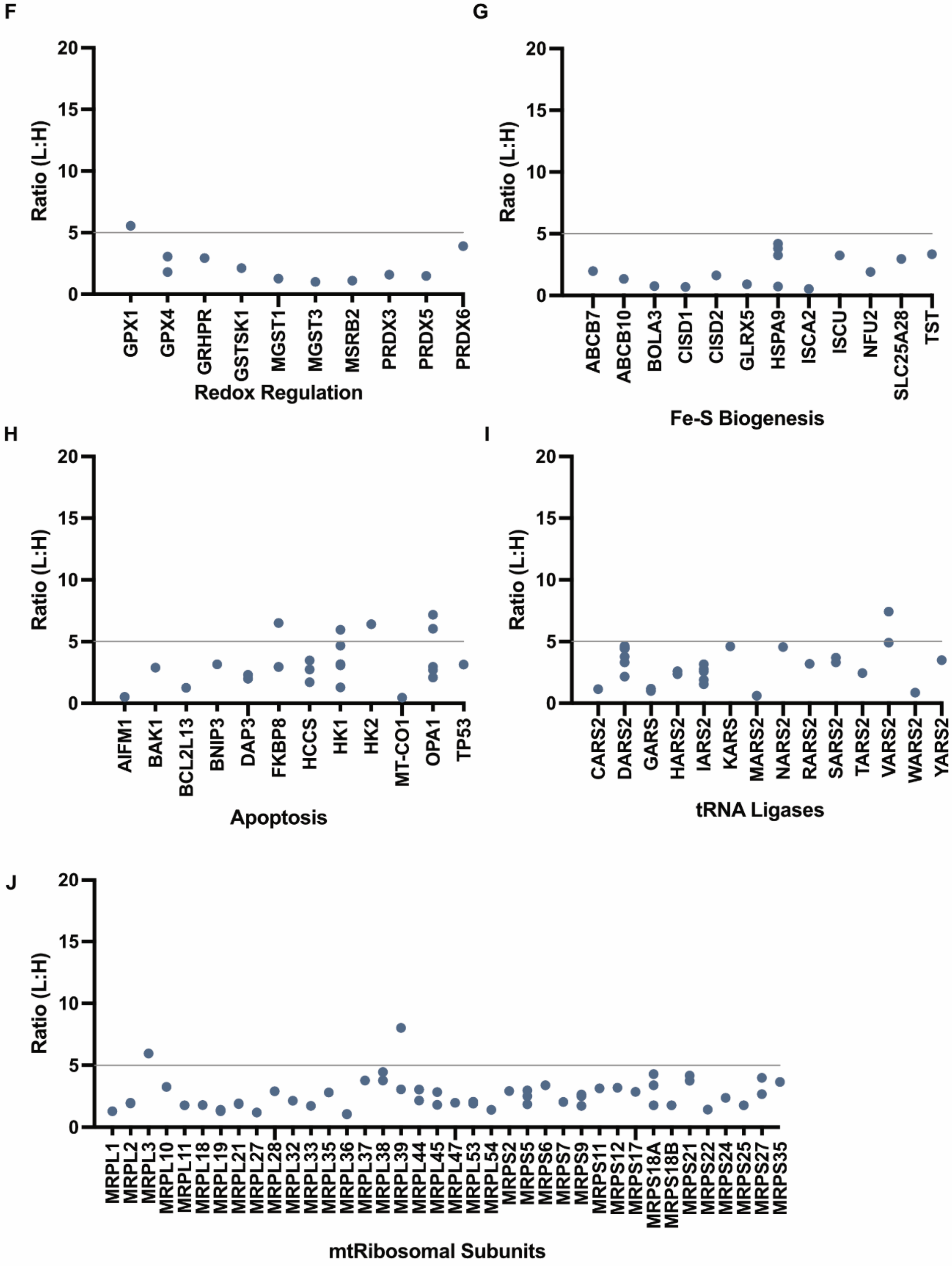

F
Ratio (L:H)
20
15
10
5
0
GPX1
GPX4
GRHPR
GSTSK1
MGST1
MGST3
MSRB2
PRDX3
PRDX5
PRDX6
Redox Regulation
G
Ratio (L:H)
20
15
10
5
0
ABCB7
ABCB10
BOLA3
CISD1
CISD2
GLRX5
HSPA9
ISCA2
ISCU
NFU2
SLC25A28
TST
Fe-S Biogenesis
H
Ratio (L:H)
20
15
10
5
0
AIFM1
BAK1
BCL2L13
BNIP3
DAP3
FKBP8
HCCS
HK1
HK2
MT-CO1
OPA1
TP53
Apoptosis
I
Ratio (L:H)
20
15
10
5
0
CARS2
DARS2
GARS
HARS2
IARS2
KARS
MARS2
NARS2
RARS2
SARS2
TARS2
VARS2
WARS2
YARS2
tRNA Ligases
J
Ratio (L:H)
20
15
10
5
0
MRPL1
MRPL2
MRPL3
MRPL10
MRPL11
MRPL18
MRPL19
MRPL21
MRPL27
MRPL28
MRPL32
MRPL33
MRPL35
MRPL36
MRPL37
MRPL38
MRPL39
MRPL44
MRPL45
MRPL47
MRPL53
MRPL54
MRPS2
MRPS5
MRPS6
MRPS7
MRPS9
MRPS11
MRPS12
MRPS17
MRPS18A
MRPS18B
MRPS21
MRPS22
MRPS24
MRPS25
MRPS27
MRPS35
mtRibosomal Subunits

K

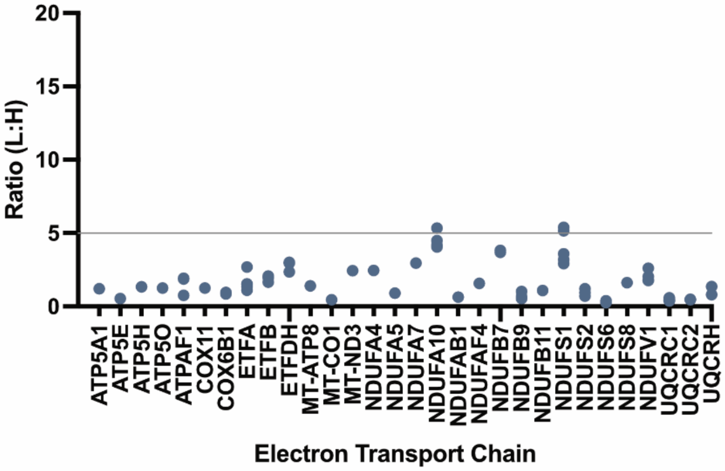

Ratio (L:H)
20
15
10
5
0
ATP5A1
ATP5E
ATP5H
ATP5O
ATPAF1
COX11
COX6B1
ETFA
ETFB
ETFDH
MT-ATP8
MT-CO1
MT-ND3
NDUFA4
NDUFA5
NDUFA7
NDUFA10
NDUFAB1
NDUFAF4
NDUFB7
NDUFB9
NDUFB11
NDUFS1
NDUFS2
NDUFS6
NDUFS8
NDUFV1
UQCRC1
UQCRC2
UQCRH
Electron Transport Chain

L

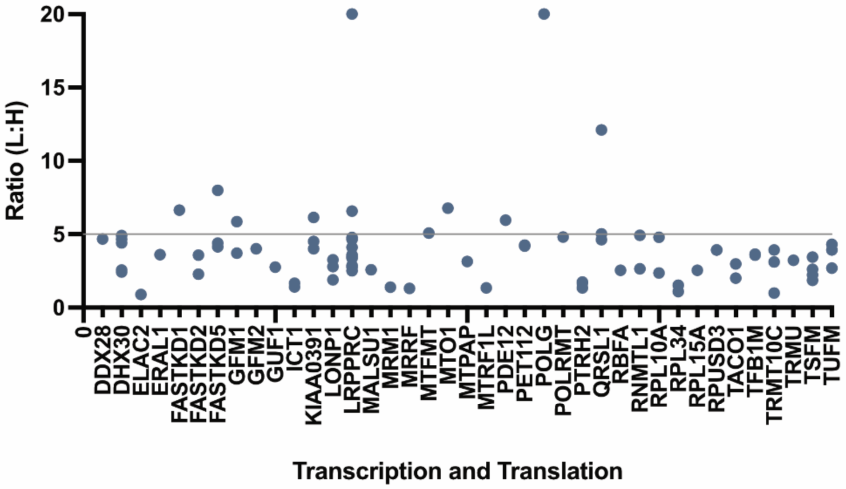

Ratio (L:H)
20
15
10
5
0
0
DDX28
DHX30
ELAC2
ERAL1
FASTKD1
FASTKD2
FASTKD5
GFM1
GFM2
GUF1
ICT1
KIAA0391
LONP1
LRPPRC
MALSU1
MRM1
MRRF
MTFMT
MTO1
MTPAP
MTRF1L
PDE12
PET112
POLG
POLRMT
PTRH2
QRSL1
RBFA
RNMTL1
RPL10A
RPL34
RPL15A
RPUSD3
TACO1
TFB1M
TRMT10C
TRMU
TSFM
TUFM
Transcription and Translation

Figure S4. L:H ratios plotted from isoTOP-ABPP analysis of 10 mM peroxide treated Mito-P samples. Identified unique cysteine residues are plotted for proteins belonging to mitochondrial biological pathways and categories including the (A) citric acid cycle, (B) innate immunity, (C) transport, (D) urea cycle, (E) fatty acid oxidation, (F) redox regulation, (G) Fe-S biogenesis, (H) apoptosis, (I) tRNA ligases, (J) mitochondrial (mt)-ribosomal subunits, (K) electron transport chain, (L) transcription and translation.

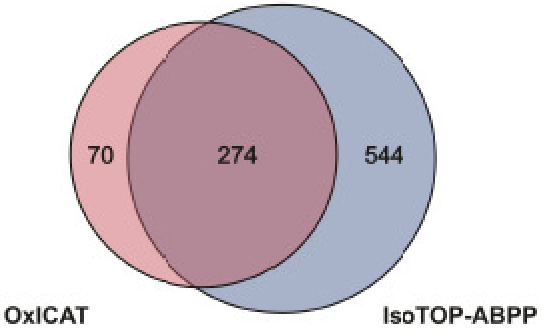

Figure S5. Overlap between isoTOP-ABPP analysis and OxICAT analysis for all identified cysteines.
